# Black Jaundice

**Published:** 1894-08

**Authors:** 


BLACK JAUNDICE.—The disease variously known as "Malarial Hæmaturia," "Malarial Hæmatinuria," "Pernicious Fever," and

"Black Jaundice" is, as is well known, very fatal. The profession are at sea as to its real nature, and know little as to its pathology; hence the great diversity of treatment. In the present state of knowledge of the disease, all treatment is empirical, more or less. The paper, by Dr. Oates, in our last issue, has attracted much attention, and the treatment there outlined, and which, he says, he has found successful, appears to be rational. But until the cause of the disease—vaguely said to be "malarial" —is ascertained; until the pathology of the disease is carefully studied, with the aid of the microscope and other laboratory accessories, we can never hope to deal intelligently with it.

In this connection the JOURNAL is authorized to say that Prof. A. J. Smith, the Pathologist of the Medical Department of the University of Texas, who is now sojourning at San Antonio, desires to study the disease, and if any of our readers within a day's travel of Galveston or San Antonio, will wire him when next a case occurs in their practice, and *before any quinine is administered*, he will take great pleasure in going to see it, and will appreciate the opportunity thus offered him, as a marked professional courtesy.

